# Non-Invasive O-Toluidine Monitoring during Regional Anaesthesia with Prilocaine and Detection of Accidental Intravenous Injection in an Animal Model

**DOI:** 10.3390/metabo12060502

**Published:** 2022-05-31

**Authors:** Beate Brock, Patricia Fuchs, Svend Kamysek, Udo Walther, Selina Traxler, Giovanni Pugliese, Wolfram Miekisch, Jochen K. Schubert, Phillip Trefz

**Affiliations:** 1 Rostock Medical Breath Research Analytics and Technologies (ROMBAT), Department of Anaesthesiology and Intensive Care Medicine, Rostock University Medical Centre, 18057 Rostock, Germany; patricia.fuchs@med.uni-rostock.de (B.B.); patricia.fuchs@uni-rostock.de (P.F.); svend.kamysek@lkros.de (S.K.); s.traxler@web.de (S.T.); g.pugliese@mpic.de (G.P.); wolfram.miekisch@uni-rostock.de (W.M.); jochen.schubert@uni-rostock.de (J.K.S.); 2 Ambulance and Rescue Service, Rostock District Administration, Mecklenburg-Vorpommern, 18209 Bad Doberan, Germany; 3 Institute of Pharmacology and Toxicology, Rostock University Medical Centre, 18057 Rostock, Germany; udo.walther@med.uni-rostock.de; 4 Max Planck Institute for Chemistry, 55128 Mainz, Germany

**Keywords:** breath gas analysis, drug monitoring, o-toluidine, prilocaine, PTR-ToF-MS, regional anaesthesia, toxicology, volatile organic compounds (VOC)

## Abstract

Regional anaesthesia is well established as a standard method in clinical practice. Currently, the local anaesthetics of amino-amide types such as prilocaine are frequently used. Despite routine use, complications due to overdose or accidental intravenous injection can arise. A non-invasive method that can indicate such complications early would be desirable. Breath gas analysis offers great potential for the non-invasive monitoring of drugs and their volatile metabolites. The physicochemical properties of o-toluidine, the main metabolite of prilocaine, allow its detection in breath gas. Within this study, we investigated whether o-toluidine can be monitored in exhaled breath during regional anaesthesia in an animal model, if correlations between o-toluidine and prilocaine blood levels exist and if accidental intravenous injections are detectable by o-toluidine breath monitoring. Continuous o-toluidine monitoring was possible during regional anaesthesia of the cervical plexus and during simulated accidental intravenous injection of prilocaine. The time course of exhaled o-toluidine concentrations considerably differed depending on the injection site. Intravenous injection led to an immediate increase in exhaled o-toluidine concentrations within 2 min, earlier peak and higher maximum concentrations, followed by a faster decay compared to regional anaesthesia. The strength of correlation of blood and breath parameters depended on the injection site. In conclusion, real time monitoring of o-toluidine in breath gas is possible by means of PTR-ToF-MS. Since simulated accidental intravenous injection led to an immediate increase in exhaled o-toluidine concentrations within 2 min and higher maximum concentrations, monitoring exhaled o-toluidine may potentially be applied for the non-invasive real-time detection of accidental intravenous injection of prilocaine.

## 1. Introduction

Regional anaesthesia is well established as a standard method in clinical practice. Currently, local anaesthetics of the amino-amide type such as lidocaine, prilocaine, mepivacaine, ropivacaine and bupivacaine are frequently used. Unwanted and unexpected side effects are most likely associated with accidental intravascular injection, overdose or the rapid absorption of local anaesthetics [1]. For all local anaesthetics, maximal doses have been recommended to minimise the risk of adverse or toxic reactions. Unfortunately, these doses do not take into account patient age, co-morbidities and co-medications. For prilocaine, the recommended maximum dose is 600 mg and 8.5 mg·kg^−1^.

The local anaesthetic prilocaine is widely used due to its low systemic toxicity, rapid onset and medium duration of action. Prilocaine (*N*-(2)-Methylphenyl-(2)-propylamino-propanamide) is associated with the formation of methaemoglobin (MetHb). Prilocaine is mainly eliminated by hepatic metabolism. Extrahepatic metabolism to a minor extent has been described [2,3,4]. The main metabolite o-toluidine (2-Methylaniline) is responsible for the formation of MetHb by oxidation of iron in haemoglobin. MetHb is of toxicological relevance, as it does not bind and transport oxygen.

To enable personalised patient care, drug monitoring for specific substances and metabolites is desirable to minimise negative effects or detect complications at an early stage. The non-invasive bed-side monitoring of prilocaine blood concentrations is not possible. Breath gas analysis offers great potential for non-invasive monitoring of drugs and their volatile metabolites [5]. The physicochemical properties of o-toluidine allow its detection in breath gas which we demonstrated in a pilot study [6]. However, the influence of injection site and possible correlation to blood concentrations were not investigated.

While prilocaine is routinely used in anaesthesiology, complications due to overdose or accidental intravenous injection can arise. A non-invasive method that can indicate such complications early on would be desirable. Therefore, the aim of this pilot study was to investigate the following:

(1) if o-toluidine breath gas concentrations could be monitored during regional anaesthesia;
(2) whether a correlation between o-toluidine breath concentrations and prilocaine blood concentrations exists;
(3) if accidental intravenous injection can be detected by o-toluidine monitoring in breath gas.

## 2. Results

### 2.1. Haemodynamics

After the intravenous administration of prilocaine, haemodynamic effects were observed. According to the study’s design, hypotension was treated with increasing norepinephrine doses in order to maintain a MAP of 60 mmHg during the experiment. Additionally, some animals showed cardiac arrhythmias. PIG 3 had to be resuscitated shortly after cardiac arrest.

**Figure 1:**
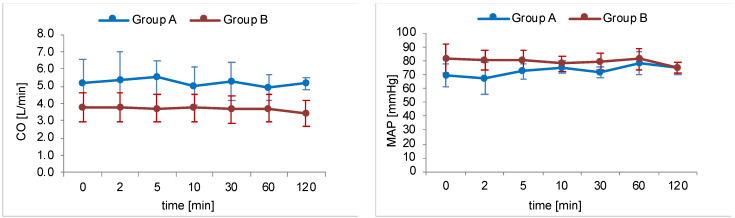
Time course of haemodynamic parameters mean arterial pressure (MAP) and cardiac output (CO). Blue lines represent group A (intravenous injection of prilocaine, *n* = 6) and red lines represent group B (cervical plexus regional anaesthesia with prilocaine, *n* = 5). Dots mark the defined measurement points as mean value of the entire group with relevant SD values.

During simulated plexus anaesthesia in group B, the pigs showed no relevant haemodynamic instability. Arrhythmias could not be observed. Blood pressure and cardiac output showed no relevant decrease except at the end of investigation. Low dose norepinephrine therapy remained constant over the experimental period in all animals. Figure 1 illustrates the time course of MAP and CO of both study groups during intervention.

### 2.2. Real-Time O-Toluidine Monitoring in Exhaled Breath

**Figure 2:**
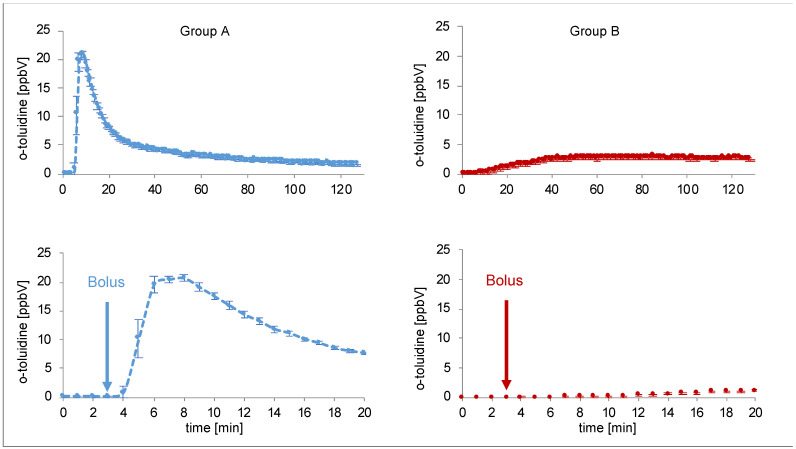
Continuous measurement of o-toluidine concentrations in breath by PTR-ToF-MS over 120 min (above) for two single pigs (blue line group A (intravenous injection of prilocaine), red line group B (cervical plexus regional anaesthesia with prilocaine)). Each data point represents mean concentrations over one minute. The diagrams at the bottom show the first 20 min. Bolus was administered at the third minute.

O-toluidine could be detected in the expired breath gas of all pigs after intervention. The time course was different depending on the type of intervention. Real time proton-transfer-reaction-time-of-flight-mass-spectrometry (PTR-ToF-MS) data are presented in Figure 2. Data from each individual pig can be found in Figures S1 and S2.

### 2.3. Time Course of Prilocaine, O-Toluidine and MetHb

**Figure 3:**
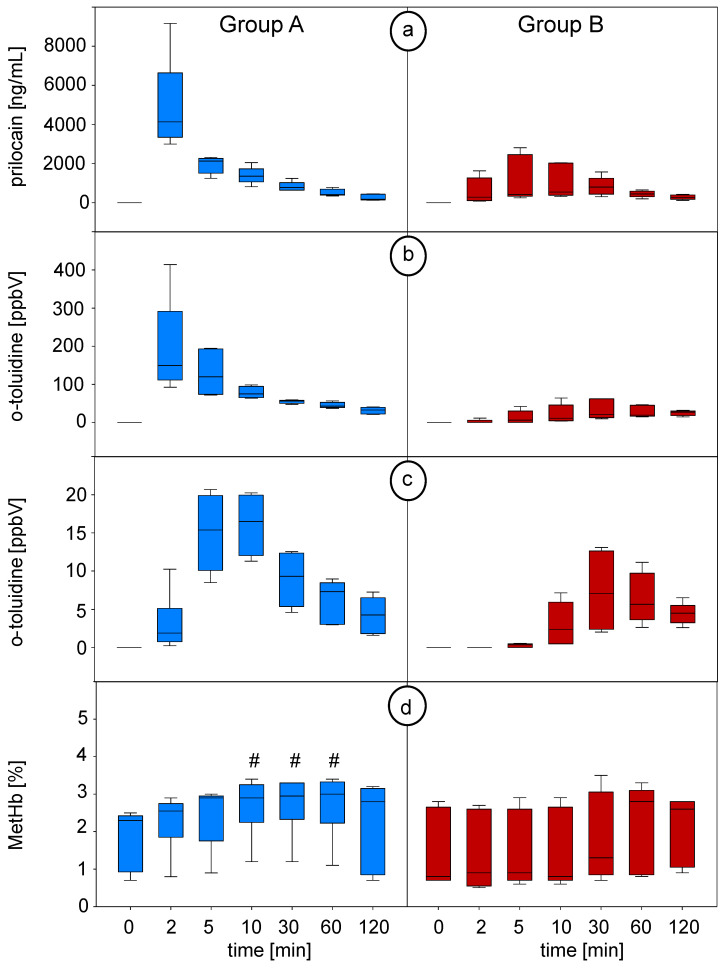
(**a**) Prilocaine concentrations in blood measured by means of GC-MS, (**b**) o-toluidine concentrations in the headspace over blood measured by means of SPME-GC-MS and (**c**) o-toluidine concentrations in breath measured by means of PTR-ToF-MS at defined timepoints (breath data from continuous measurement were averaged over the corresponding minute). (**d**) Percentage values of methaemoglobin (MetHb) related to haemoglobin (Hb) during the experiment. # indicates statistically significant changes in MetHb versus P0 (0 min) (repeated measures ANOVA on ranks, Dunn’s post hoc test and *p* < 0.05 was considered significant). Blue plots represent group A (intravenous injection of prilocaine), and red plots represent group B (cervical plexus regional anaesthesia with prilocaine). P0: before prilocaine injection; P1: 2 min; P2: 5 min; P3: 10 min; P4: 30 min; P5: 60 min; P6: 120 min after prilocaine injection.

Time courses of prilocaine in blood, o-toluidine concentrations in blood and breath and MetHb values throughout the intervention are shown in Figure 3.

Intravenous injection of prilocaine in **group A** resulted in a profound increase in prilocaine and o-toluidine concentrations in blood. Both substance concentrations reached their peak at the same time point (P1 at second minute). The following decrease in both concentrations showed an almost exponential decay. Exhaled concentrations of o-toluidine rapidly increased within the first 2 min after bolus injection with a steep slope of the curve, and it reached its peaked concentration at P3 after 10 min. Afterwards, o-toluidine breath concentrations showed an almost exponential decay.

In **group B** (plexus anaesthesia), the increase in prilocaine and o-toluidine blood concentrations occurred slower and was less profound after the infiltration of the cervical plexus. Prilocaine median blood concentrations reached their maximum after 30 min. O-toluidine concentrations in blood reached a maximum after 30 min. Exhaled concentrations of o-toluidine increased consistently after the fifth minute during regional anaesthesia. In contrast to group A, curves of o-toluidine concentrations in breath differed between the pigs, and peak concentrations were not reached before 30 min. Afterwards, the concentrations in blood and breath decreased slowly. Maximum concentrations were consistently lower in group B compared to group A. 

MetHb (percentage related to haemoglobin) increased significantly at 10–60 min in group A (from P3 after 10 min until P5 after 60 min) and reached a plateau afterward, while no significant changes occurred in group B. During the experiment, no relevant methaemoglobinemia could be observed.

### 2.4. Correlation of Blood and Breath Concentrations

We performed Pearson’s product moment correlation analysis to investigate linear relationships between prilocaine in blood, o-toluidine in the headspace of blood and o-toluidine in exhaled breath (Table S1). Correlation analysis was performed for all pigs as well as for both groups separately. In all cases, we found a significant correlation of prilocaine in blood and o-toluidine in the headspace of blood. This correlation was stronger in group A (r = 0.66) compared to group B (r = 0.47). No significant correlation was found between o-toluidine in breath and prilocaine in blood. O-toluidine in breath showed a strong correlation with o-toluidine in the headspace of blood in group B (r = 0.85) but no significant correlation in group A.

## 3. Discussion

In this pilot study, real time monitoring of o-toluidine, the main metabolite of prilocaine, was performed in the exhaled breath of mechanically ventilated pigs by means of PTR-ToF-MS. In addition, we measured prilocaine and o-toluidine concentrations in blood at defined time points. Continuous o-toluidine monitoring in breath was possible during a regional anaesthesia of the cervical plexus and during simulated accidental intravenous injections of prilocaine.

Prilocaine plasma concentrations depend on the injection site [7,8]. Consequently, different types of injection led to pronounced differences in the time course of prilocaine and o-toluidine concentrations in blood and breath. The intravenous injection of prilocaine resulted in an immediate increase and much higher peak concentrations of o-toluidine in blood and breath as well as in higher prilocaine concentrations in blood than during cervical plexus anaesthesia. Maximum concentrations were reached faster due to intravenous drug administration compared to regional anaesthesia with prilocaine.

Time course and drug concentration levels in blood depend, furthermore, on dose, speed of injection, tissue perfusion, pharmacological properties of the drug, distribution and compartmental redistribution. The intravenous injection of a drug leads to an immediate increase in blood concentration, followed by a fast decrease, and all in all shows a typical time course of concentration [9,10]. For a regional anaesthesia of the cervical plexus, prilocaine was infiltrated into the vascular nerve sheath. Resulting blood concentrations depend mainly on tissue perfusion and resulting absorptions. Therefore, prilocaine blood concentrations increased with a time delay, which is in agreement with previous findings [11,12]. Prilocaine and o-toluidine blood concentrations in study group B were lower compared to study group A.

As described above, intravenous injection (group A) led to an immediate increase in prilocaine and o-toluidine blood concentrations. O-toluidine concentrations in breath showed a fast increase as well but, in contrast, reached its maximum concentration later compared to blood concentrations. A steady decline could be observed subsequently. While prilocaine and o-toluidine concentrations in blood showed a significant correlation, no significant correlation could be established between prilocaine in blood and o-toluidine in breath within this group when all measured concentration data during the entire study period are taken into account.

A reasonable correlation of blood and breath concentrations requires steady-state conditions, which are not achieved immediately after bolus injection. While prilocaine and o-toluidine blood concentrations increased immediately and reached its maximum at 2 min after intravenous injection, breath concentrations of o-toluidine showed a fast and steep increase during the first 2 min, but peak breath concentrations were reached several minutes later. Additionally, the exhalation of volatile organic compounds (VOCs) strongly depends on physiologic parameters and, thus, a short period of haemodynamic instability with a moderate decrease in cardiac output within group A directly after the bolus injection of prilocaine may have further influenced o-toluidine exhalation. A comparable behaviour has already been reported for propofol [13]. In contrast, if only data from time point P3 (after 10 min) onwards are considered, when a steady state can be assumed after haemodynamic stabilization, a significant correlation (*p* < 0.001) between blood and breath o-toluidine concentrations (r = 0.72) as well as between o-toluidine breath and prilocaine blood concentrations (r = 0.67) can be established.

In addition to physiological and pharmacological aspects, the contribution of the analytical system on substance concentrations has to be discussed. O-toluidine is an aromatic amine and, thus, a reactive compound. Such compounds tend to react with the tubing materials of the analytical system and response time can be delayed. While our system is optimised for breath analysis [14], further optimizations of the measurement conditions and inlet system could reduce possible interactions of o-toluidine with the surfaces within the analytical system [15].

In the regional anaesthesia group B, prilocaine and o-toluidine blood concentrations as well as o-toluidine breath concentrations reached their peak median concentrations after 30 min and slowly decreased afterward. O-toluidine blood and breath concentrations were well correlated (r = 0.85), whereas the correlation of blood o-toluidine and prilocaine in blood was weaker compared to group A. As described above, prilocaine blood concentrations were considerably lower after local injection for cervical plexus anaesthesia due to slow absorption. The systemic distribution of prilocaine was much slower compared to intravenous injection, and hemodynamic parameters were within a normal range. Thus, a steady state can be assumed, resulting in a better agreement of o-toluidine blood and breath concentrations. Additionally, minor effects from the analytical system regarding response time will be neglectable in this scenario due to the overall slower dynamics.

Although regional anaesthesia with the local anaesthetic prilocaine is a routine method in anaesthesiology, complications due to overdose or accidental intravenous injection are described. Cardiac and cerebral toxicity are of clinical relevance and can be life-threatening depending on the resulting blood concentrations.

High prilocaine blood levels cause methaemoglobinemia, as the main metabolite o-toluidine induces the oxidation of haemoglobin. Relevant methaemoglobinemia has been reported as a side effect for different local anaesthetics [16,17,18] and especially for prilocaine [19,20,21]. Most frequently affected groups include children and less often involve patients with proven glucose-6-phosphate dehydrogenase deficiency, hemoglobinopathies or methaemoglobin-promoting medication [22,23,24]. The early diagnosis of methaemoglobinemia by means of blood gas analysis is essential for the initiation of adequate therapy. In this pilot study, MetHb (percentage related to haemoglobin) increased in group A, while no significant changes occurred in group B. The administered prilocaine doses in the experiment did not cause relevant methaemoglobinemia in pigs.

Regarding local anaesthetic toxicity due to high prilocaine blood levels, overdose or accidental intravenous injection have to be avoided. The accurate and careful execution of regional-anaesthesia techniques (e.g., slow fractionated injection technique including aspiration tests), adherence to maximum dose and application of ultrasound-guided techniques decrease the above-mentioned risks. These preventive measures according to guidelines are clinical standards and mandatory in order to ensure patient safety. Real-time monitoring of exhaled o-toluidine may potentially be applied for the early non-invasive detection of accidental intravenous injection of prilocaine. An additional non-invasive analytical method that can indicate rapidly increasing blood concentrations due to the accidental injection of prilocaine might be helpful as an early warning tool and could facilitate stopping injection immediately. Thereby, patient safety can be increased, even if no direct correlation of blood prilocaine and breath o-toluidine can be established during the first minutes of injection. Since prilocaine absorption depends on the site of regional anaesthesia, sites other than cervical plexus should also be assessed in further studies to describe the time course of o-toluidine concentrations in breath in the perspective of future human clinical applications.

For routine applications in clinical practice, the development of cheap and simple to use point-of-care sensor technology would be mandatory to replace the complex and expensive equipment used in this study.

In conclusion, real time monitoring of the main prilocaine metabolite o-toluidine in breath gas is possible by means of PTR-ToF-MS. This pilot study contributes to the growing evidence that PTR-ToF-MS can be used for basic research in metabolic drug monitoring of volatile metabolites. Regional anaesthesia and simulated accidental intravenous injection of prilocaine resulted in a marked difference of the time course and level of exhaled o-toluidine concentrations, but no direct correlation of blood prilocaine and breath o-toluidine concentrations could be established initially.

Simulated accidental intravenous injection consistently led to an immediate increase of o-toluidine breath concentrations within the first two minutes, a characteristic steeper slope of the curve and higher maximum concentrations compared to plexus regional anaesthesia. Therefore, the applied monitoring of exhaled o-toluidine may potentially be established for early non-invasive detections of accidental intravenous prilocaine injections.

## 4. Material and Methods

### 4.1. Ethical Proposal

After approval by the Local Animal Ethics Committee (State Office for Agriculture, Food Safety and Fishery in Mecklenburg-Vorpommern, Germany; reference number: LAALF MV 7221.3-1.1-022/18, date of permission 22 May 2018), an animal model was developed. All procedures were carried out in accordance to relevant laws and guidelines.

### 4.2. Animals

Eleven male pigs (German Landrace) with a body weight between 35 and 61 kg (mean 45 ± 8 kg) were included in the study.

### 4.3. Anaesthesia and Instrumentation

After overnight fasting and receiving water ad libitum, all experimental animals were given an intramuscular premedication with 10 mL azaperone (40 mg/mL), 15 mL ketamine (50 mg/mL) and 1.5–2 mL midazolam (5 mg/mL). Standard monitoring (electrocardiogram and oxygen saturation) was implemented (Datex Ohmeda GmbH, Duisburg, Germany). An arterial cannula was placed in a femoral artery to measure blood pressure after the induction of anaesthesia. A peripheral venous cannula was inserted in an ear vein, and anaesthesia was induced by a bolus injection of 1–2 µg·kg^−1^ fentanyl (Fentadon^®^, Eurovet Animal Health B.V., Bladel, the Netherlands), 2 mg·kg^−1^ propofol 2% (Propofol Lipuro^®^, B. Braun Melsungen AG, Melsungen, Germany) and 0.1–0.15 mg·kg^−1^ cis-atracurium (Nimbex^®^ 10 mg, GlaxoSmithKline GmbH & Co. KG, Munich, Germany). After pre-oxygenation, mask ventilation pigs were intubated orally with a tracheal tube (ID 8.0 mm, Mallinckrodt, Hazelwood, MO, USA) and pressure-controlled ventilation with FiO_2_ 0.4 (Servo 300^®^—ventilator, Siemens, Erlangen, Germany) was carried out. Basic parameters were as follows: tidal volume of 8–10 mL·kg^−1^, positive end-expiratory pressure of 5 cmH_2_O and a respiratory rate between 14 and 20 min ^1^. The adjustment of ventilation was carried out using end-tidal carbon dioxide (p_et_CO_2_) measurements and arterial blood gas analysis to maintain normoventilation aiming for 4.5–5.5 kPa paCO_2_. The ventilatory settings were then kept constant during the experiment. Anaesthesia was maintained by a continuous infusion of 6–8 mg·kg^−1^·h^−1^ propofol, 0.05–0.1 mg·kg^−1^·h^−1^ midazolam, 0.05–0.2 mg·kg^−1^ cis-atracurium and 1–2 µg·kg^−1^·h^−1^ fentanyl.

The initial instrumentation was extended by a tri-lumen central jugular venous catheter (Arrow International Inc., Reading, PA, USA). Continuous invasive blood pressure and hemodynamic monitoring were carried out via the initially placed PiCCO^®^-catheter, inserted into the femoral artery.

Normovolemia was ensured by a continuous administration of 6–10 mL·kg^−1^·h^−1^ crystalloid solution (Elektrolyt-Infusionslösung 153^®^, Serumwerk Bernburg AG, Bernburg, Germany). Fluid management was adapted to blood pressure and hemodynamic parameters were measured by the PiCCO^®^-system, with the following target values: mean arterial pressure > 60 mm Hg and stroke volume variation (SVV) < 15%. After balancing volume deficits, norepinephrine (Arterenol^®^, Sanofi-Aventis GmbH, Frankfurt, Germany) was administered continuously in adequate dosages to maintain constant target mean arterial blood pressure.

### 4.4. Study Design

After the induction of anaesthesia and completion of monitoring, the animals were assigned to the following procedures, resulting in six pigs being assigned to group A and five pigs to group B.

**Group A: Simulation of an accidental intravenous injection of prilocaine during regional anaesthesia**. Pigs received an intravenous injection of prilocaine (Xylonest^®^ 2%) in a dosage of 300–400 mg (15–20 mL) corresponding to 5.8–7.2 mg·kg^−1^ body weight. In order to ensure intravenous administration, the substance was injected via the central venous catheter.

**Group B: Simulation of cervical plexus regional anaesthesia using prilocaine**. An ultrasound-guided infiltration of plexus cervicalis with prilocaine (Xylonest^®^ 2%) in a dosage of 300 mg (=15 mL) was carried out. The prilocaine dose corresponded to 7.3–8.5 mg·kg^−1^ body weight. To avoid intravascular injection, infiltration was monitored continuously by ultrasound and additional aspiration control with a syringe was performed.

Breath gas analysis was carried out in real-time modes by means of PTR-ToF-MS. In addition, breath and blood samples were taken simultaneously at defined time points (P0-P6). Blood samples were analysed using two different analytical methods: the detection of o-toluidine by means of headspace-solid phase microextraction coupled with gas chromatography mass spectrometry (HS-SPME-GC-MS) over blood and the detection of applicated prilocaine by means of gas chromatography mass spectrometry (GC-MS).

**Figure 4:**
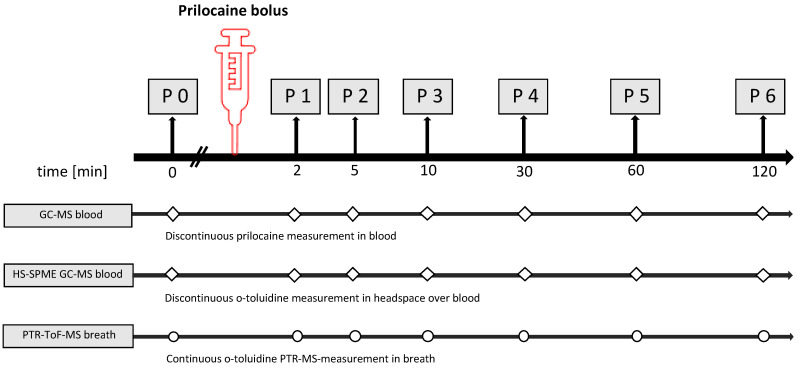
Schedule of the experimental procedures for both study groups (group A: intravenous injection group B: regional anaesthesia/infiltration). Discontinuous measurements in blood (squares) were carried out for prilocaine by GC-MS and for o-toluidine in the headspace over blood by HS-SPME-GC-MS. Continuous monitoring of breath gas was performed by PTR-TOF-MS (dots).

Blood examinations, hemodynamic and respiratory parameter measurements and arterial blood gas analyses were performed simultaneously. Breath gas was analysed continuously. A graphical study protocol is shown in Figure 4.

### 4.5. Analytical Measurements and Data Assessment

#### 4.5.1. Haemodynamic Measurements

Mean arterial pressure (MAP), heart rate (HR), stroke volume variation (SVV) and cardiac output (CO) were determined by the PiCCO^®^-system (5 F, 20 cm, thermodilution catheter PV2015L20-46N, Pulsion Medical Systems, Feldkirchen, Germany) and placed in the pig‘s femoral artery. Measurements of cardiac output were carried out by using a thermodilution technique (mean of three injections of 15 mL ice-cold saline via central venous catheter) for all discontinuous measurements (at time points P0: before prilocaine injection, P1: 2 min, P2: 5 min, P3: 10 min, P4: 30 min, P5: 60 min and P6: 120 min after prilocaine injection, respectively).

#### 4.5.2. Blood Gas Analysis

Arterial blood samples of 1 mL were taken into a special tube loaded with lithium-heparin (Monovette^®^, Luer, 1 mL LH, Sarstedt AG & Co. KG, Nürnbrecht, Germany). Haemoglobin, methaemoglobin-fraction, partial pressure of carbon dioxide (p_a_CO_2_) and oxygen (p_a_O_2_); acid base status; electrolytes; and lactate and glucose concentrations were measured by an autoanalyser (ABL 800^®^, Radiometer GmbH, Krefeld, Germany).

#### 4.5.3. Blood Examination/Toxicology

In addition to breath gas monitoring (see Section 4.5.4), prilocaine blood concentrations were determined by GC-MS, and the concentrations of the main metabolite o-toluidine in the headspace over blood were measured by means of SPME-GC-MS.

##### Discontinuous Prilocaine Detection in Blood

For the determination of prilocaine in blood, 3 mL arterial blood was withdrawn in serum tubes (S-Monovette^®^, 4.5 mL, coagulation activator, Sarstedt AG & Co.KG, Nürnbrecht, Germany). After resting for 30 min in an upright position, samples were centrifuged for 10 min at room temperature, and the supernatant transferred to an Eppendorf^®^ tube (Eppendorf AG, Hamburg, Germany) and frozen at −20 °C until toxicological examination after two weeks. For the calibration samples, prilocaine was obtained from AstraZeneca^®^ as an injection solution with 8.579 g/L prilocaine.

Prilocaine concentrations were measured by gas chromatography mass spectrometry on an HP 5890 equipped with an HP 5971 MS detector, and mepivacaine (final concentration 3.75 µg/mL) was used as the internal standard. The substances were quantified by their masses of 86 (prilocaine) and 98 (mepivacaine), respectively. The linearity of the method was between 10 ng/mL and 1 µg/mL, the sensitivity was tested for <1 ng/mL, and the specificity of prilocaine was confirmed in all samples by the ratio of the masses 86 and 44. Injection temperature was 280 °C, and a furnace temperature gradient between 70 and 300 °C and a MS–detector interface temperature of 280 °C were used for the analysis by an HP-1MS column (Agilent Technologies Deutschland GmbH, Waldbronn). The MSD was operated in scan mode (detected masses between 40 and 450).

Prior to GC analysis, the samples were diluted with human plasma in a ratio of 1:2, 1:5 or 1:10 (same dilution ratio for a series of samples from the same animal) to reach the linear concentration range and to obtain a very similar matrix as the standard samples. The samples were alkalised by adding one fifth of the sample volume of a carbonate buffer (0.8 mol/L, pH 9.3) and then subjected to liquid–liquid extraction (chloroform: acetonitrile: ethyl acetate as 4:3:2) with, again, a fifth of the sample volume. A solvent extract measuring 1 µL was injected into GC.

Prilocaine was measured in our samples by GC/MS according to validated methods [25,26] with minor modifications by at least a seven-point calibration curve between 5 and 300 ng/mL or 20 and 1500 ng/mL. Measurements took place in five series (different days) after samples were diluted with human plasma in ratios of 1:2 up to 1:10 to fit into the calibration curves (each pig series with the same dilution ratio). The largest difference between spiked and calculated values of calibration samples was found with the 20 ng/mL concentration with 92.2% (±7.7%, *n* = 5; relative standard deviation (RSD): 8.3%), and all other differences were calculated between 93.9% (±5.5%; 50 ng/mL, RSD: 5.9%, *n* = 3) and 103.7% (±8.5%; 800 ng/mL, RSD: 8.2%, *n* = 3). The limit of detection can be found in Table 1).

##### Discontinuous Measurement of O-Toluidine in the Headspace over Blood by Means of HS-SPME-GC-MS

The main metabolite of prilocaine, o-toluidine, was determined in the headspace over arterial blood by means of HS-SPME-GC-MS measurements. Therefore, full blood was withdrawn in Li-Heparin-tubes (S-Monovette^®^, 2.7 mL, Lithium-Heparin, Sarstedt AG & Co. KG, Nürnbrecht, Germany), and 1 mL was transferred into 20 mL headspace vials filled with 3 mL phosphate buffer to prevent coagulation. The vials were closed with Teflon-coated rubber septa in combination with magnetic crimp caps (Gerstel GmbH & Co. KG (Mülheim/Ruhr, Germany). Preconcentration was performed by means of Carboxen/polydimethylsiloxane (CAR/PDMS)-SPME-fibres (75 µm, SIGMA, Bellefonte, PA, USA) using a CombiPAL autosampler (CTC analytics AG, Zwingen, Switzerland) with 3 min equilibration time at 42 °C and 7 min adsorption time. For the separation and detection of o-toluidine desorbed from the SPME fibre, a GC-MS system (Agilent 7980A/5975C inert XL MSD, Santa Clara, CA, USA) was used as described before [27]. Quantification was performed via 5-point calibration in the range of 66.3 to 1060.5 ppbV (0.32–5.07 ug/mL). Two replicates were analysed per concentration level. Relative standard deviation was 4.89% at 530.2 ppbV (*n* = 4) and calculated value was 99.0% (±4.83%) of spiked value. The limit of detection was determined according to standard practices from the analysis of blank samples (*n* = 10) (see Table 1).

#### 4.5.4. Breath Gas Monitoring

##### Online Monitoring of End-Tidal CO_2_

The measurements of end-tidal CO_2_ were carried out by means of side stream capnometry (Datex Ohmeda GmbH, Duisburg, Germany).

##### Online Measurements of O-Toluidine in Breath by Means of PTR-ToF-MS

For continuous measurements of o-toluidine in breath, we used a PTR-ToF-MS 1000^®^ (Ionicon Analytik GmbH, Innsbruck, Austria). The working principle and conditions of the instrument for breath sampling have been described in previous publications [14,28]. Breath sampling was performed using a 6 m long heated silico–steel transfer line (ID 0.75 mm, Restek, Bellafonte, PA, USA). The transfer line temperature was 75 °C. Air was sampled continuously via a sterilised T-piece in side-stream mode. The sampling flow was 20 sccm. The drift tube pressure was 2.2 mbar, the drift voltage was 606 V and the drift temperature was 75 °C, resulting in an E/N ratio of 138 Td. The time resolution was 200 ms. Data were processed using PTR-MS Viewer v. 3.2.8 software (Ionicon Analytik GmbH, Innsbruck, Austria). O-toluidine was identified using its protonated monomer at *m/z* 108.081 and isotopic patterns. The ion abundance was measured in counts per second (cps), and to account for possible variations of the reagent ion signals, measured ion intensities were normalised to H_3_O^+^ counts. Expiratory and inspiratory phases of each breath were recognized by means of a Matlab-based algorithm called “breath tracker” (MATLAB version 7.12.0.635, R2011a). Every 200 ms, a complete mass spectrum was recorded. PTR-ToF-MS data (in counts per second) from breath measurements were processed by means of a custom made Matlab-based algorithm [14]. Needle trap micro extraction coupled with GC-MS was applied as previously described to verify the presence of o-toluidine in exhaled breath [27,29]. O-toluidine in exhaled breath was quantified via 4-point calibration with pure reference substance in the range of 1–100 ppbV. Humidified gas standards were generated by means of a liquid calibration unit (LCU, Ionicon Analytic, Innsbruck, Austria). Relative standard deviation was 0.67% at 100 ppbV (*n* = 3) and the calculated value was 97.0% (±0.65%) of spiked value. The limit of detection determined via signal-to-noise (S/N) from blank measurements (*n* = 10) was 0.09 ppbV (S/N 3). Table 1 summarises the analytical parameters for prilocaine detection in blood by means of GC-MS, o-toluidine detection in breath by means of PTR-ToF-MS and o-toluidine detection in the HS over blood by means of SPME-GC-MS.

#### 4.5.5. Statistics

Statistical analyses were performed using SigmaPlot 14.0 (Systat Software GmbH, Erkrath, Germany). For the identification of statistically significant differences between MetHb values in blood, a Friedman repeated measures ANOVA on ranks test with Dunn’s post hoc test was performed. A value of *p* < 0.05 was considered as statistically significant.

In order to investigate linear relationships between prilocaine in blood, o-toluidine in the headspace of blood and o-toluidine in exhaled breath, we performed Pearson’s product moment correlation analysis. Correlation analysis was performed for all pigs as well as for groups A+B separately.

## Figures and Tables

**Table 1:** Analytical parameters for prilocaine detection in blood by means of GC-MS, o-toluidine detection in breath by means of PTR-ToF-MS and o-toluidine detection in the HS over blood by means of SPME-GC-MS. LOD = limit of detection; LOQ = limit of quantification; R^2^ = correlation coefficient of calibration curve.

Analytical Parameter	Calibration R^2^	LOD (3 SD)	LOQ (10 SD)
Prilocaine calibration in blood by GC-MS in ng/mL	0.99	1.1	2.3
o-toluidine calibration in breath by PTR-ToF-MS in ppbV	0.99	0.1	0.2
o-toluidine calibration in HS over blood by SMPE-GC-MS in ppbV	0.99	2.8	3.3

## Data Availability

The data presented in this study are available upon request from the corresponding author.

## Supplementary Materials

The following supporting information can be downloaded at: https://www.mdpi.com/article/10.3390/metabo12060502/s1, Figure S1: Continuous measurement of o-toluidine concentrations in breath by PTR-ToF-MS over 120 min for six single pigs from Group A (intravenous injection of prilocaine, blue line, diagrams on the left side), Figure S2: Continuous measurement of o-toluidine concentrations in breath by PTR-ToF-MS over 120 min for five single pigs from Group B (cervical plexus regional anaesthesia with prilocaine, red line, diagrams on the left side). Each data point represents mean concentrations over one minute. Table S1: Pearson product correlation coefficients between blood and breath parameters (P1–P6).
