# Supplementary material for: Non-Invasive O-Toluidine Monitoring during Regional Anaesthesia with Prilocaine and Detection of Accidental Intravenous Injection in an Animal Model
Source: Metabolites. 2022 May 31;12(6):502. doi: 10.3390/metabo12060502 (PMC9229214; doi:10.3390/metabo12060502)
Supplement: Supplementary file 1 [file metabolites-12-00502-s001.zip › metabolites-1697782-supplementary.pdf]

metabolites

Supplement Figures S1 and S2 and Table S1:

Pigs Group A (Intravenous injection of prilocaine during regional anaesthesia)

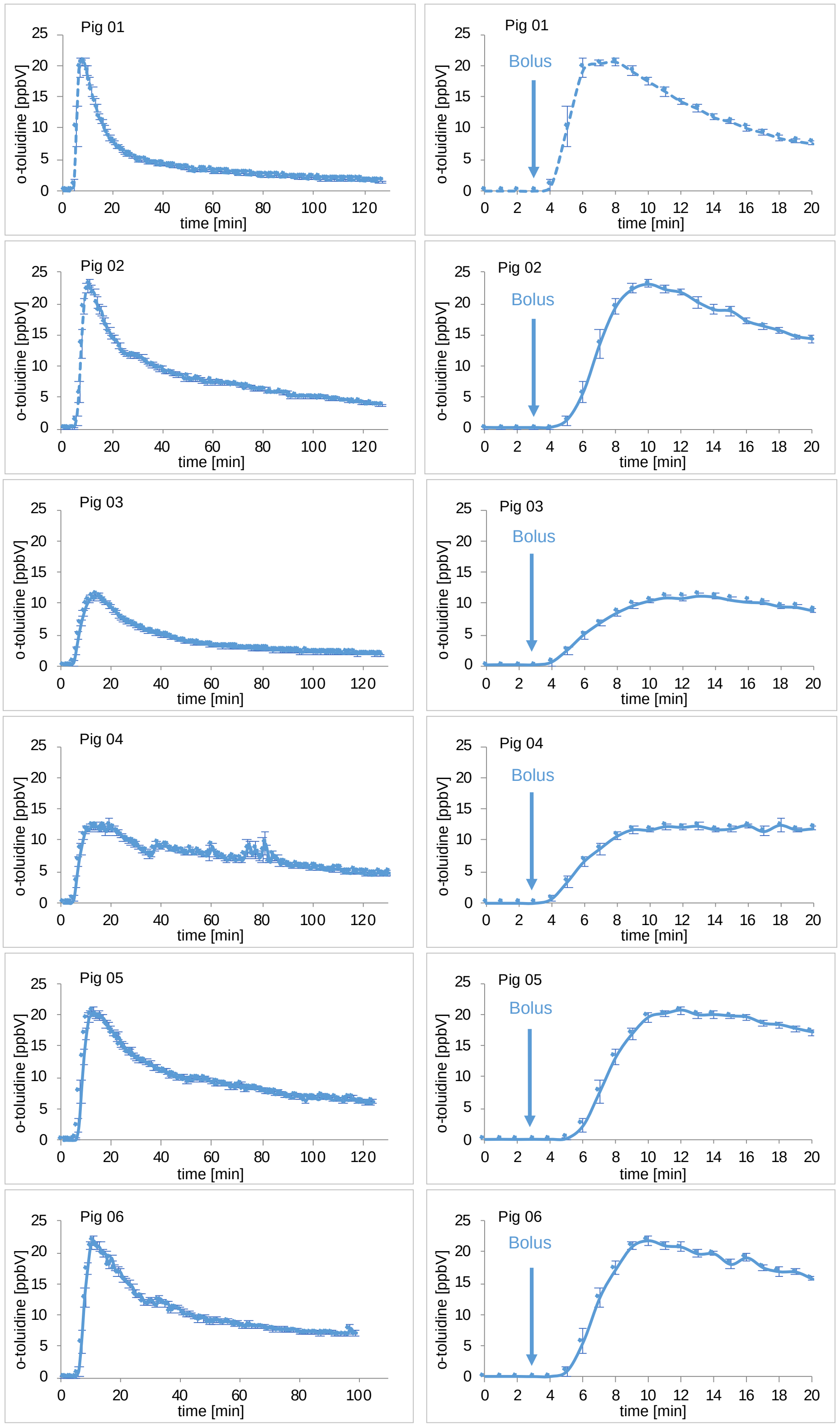

Figure S1. Continuous measurement of o-toluidine concentrations in breath by PTR-ToF-MS over 120 min for six single pigs from Group A (intravenous injection of prilocaine, blue line, diagrams on the left side). Each data point represents mean concentrations over one minute. The diagrams on

the right side show o-toluidine breath concentrations during the first 20 min. Bolus was administered at 3rd minute.

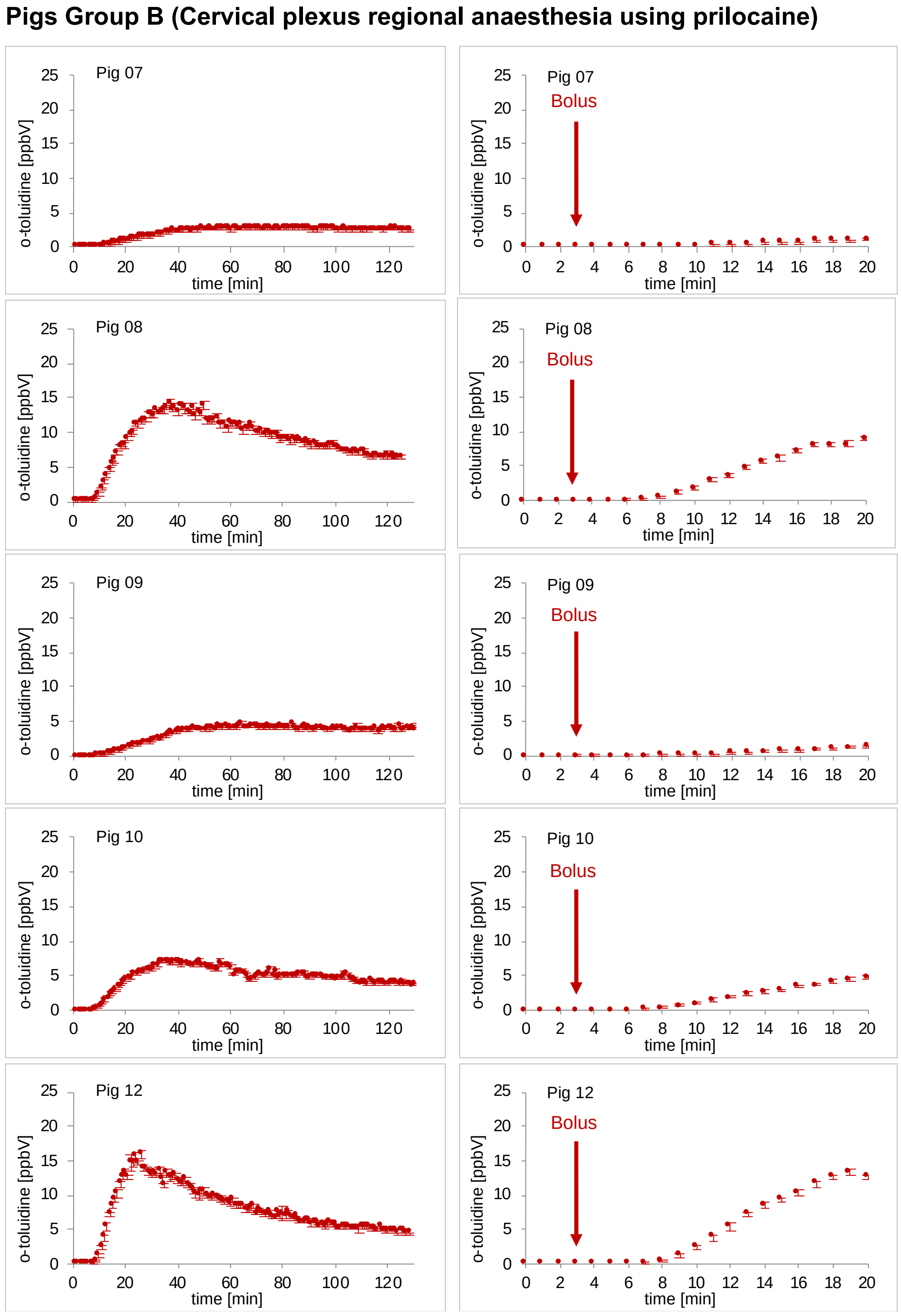

.

Figure S2. Continuous measurement of o-toluidine concentrations in breath by PTR-ToF-MS over 120 min for five single pigs from Group B (cervical plexus regional anaesthesia with prilocaine, red line, diagrams on the left side). Each data point represents mean concentrations over one minute. The diagrams on the right side show o-toluidine breath concentrations during the first 20 min. Bolus was administered at 3rd minute.

Table S1. Pearson product correlation coefficients between blood and breath parameters (P1-P6). Statistically significant correlations ($p < 0.05$) are marked in bold.

			o-toluidine in blood	o-toluidine in breath
**Both groups**	**prilocaine in blood**	**r**	**0.68**	0.033
		**p**	**<0.001**	0.80
	**o-toluidine in blood**	**r**		**0.316**
		**p**		**0.011**
**Only group A**	**prilocaine in blood**	**r**	**0.66**	-0.167
		**p**	**<0.001**	0.34
	**o-toluidine in blood**	**r**		0.015
		**p**		0.93
**Only group B**	**prilocaine in blood**	**r**	**0.47**	0.08
		**p**	**0.008**	0.66
	**o-toluidine in blood**	**r**		**0.85**
		**p**		**<0.001**
